# Role of serum interleukin-6 in deciding therapy for multidrug resistant oral lichen planus

**DOI:** 10.4317/jced.52376

**Published:** 2015-10-01

**Authors:** Sinny Goel, Akanksha Marwah, Smita Kaushik, Vijay K. Garg, Sunita Gupta

**Affiliations:** 1 MDS, Postgraduate student, Dept. of Oral Medicine and Radiology, Maulana Azad Institute of Dental Sciences, Delhi; 2 MSc. Research associate, Dept. of Oral Medicine and Radiology, Maulana Azad Institute of Dental Sciences, Delhi; 3 MD, Professor, Dept. of Biochemistry, Maulana Azad Medical College, Delhi; 4 MD, MNAMS, Director Professor & Head, Department of Dermatology & STD, Maulana Azad Medical College and Associated Lok Nayak Hospital; 5 MDS, MBA (HCA), FICD (USA), Professor and Head, Dept. of Oral Medicine and Radiology, Maulana Azad Institute of Dental Sciences, Delhi

## Abstract

**Background:**

Oral lichen planus (OLP) is a T cell mediated immune response. T cells locally present in the involved tissues release cytokines like interleukin-6 (IL-6), which contributes to pathogenesis of OLP. Also IL-6 has been associated with multidrug resistance protein (MRP) expression by keratinocytes. Correspondingly, upregulation of MRP was found in OLP. We conducted this study to evaluate the effects of various drugs on serum IL-6 in OLP; and correlation of these effects with the nature of clinical response and resistance pattern seen in OLP lesions with various therapeutic modalities. Thus we evaluated the role of serum IL-6 in deciding therapy for multidrug resistant OLP.

**Material and Methods:**

Serum IL-6 was evaluated in 42 erosive OLP (EOLP) patients and 10 normal mucosa and 10 oral squamous cell carcinoma cases using ELISA technique. OLP patients were randomly divided into 3 groups of 14 patients each and were subjected to Pimecrolimus local application, oral Mycophenolate Mofetil (MMF) and Methotrexate (MTX) alongwith Pimecrolimus local application. IL-6 levels were evaluated before and after treatment.

**Results:**

Serum IL-6 levels were raised above 3pg/ml in 26.19% erosive OLP (EOLP) cases (mean- 3.72±8.14). EOLP (5%) cases with IL-6 levels above 5pg/ml were resistant in MTX group. However significant decrease in serum IL-6 corresponding with the clinical resolution was seen in MMF group.

**Conclusions:**

Significantly raised IL-6 levels in EOLP reflect the chronic inflammatory nature of the disease. As serum IL-6 levels significantly decreased in MMF group, correspondingly no resistance to treatment was noted. However with MTX there was no significant decrease in IL-6 and resistance to treatment was noted in some, especially plaque type lesions. Thus IL-6 can be a possible biomarker in deciding the best possible therapy for treatment resistant OLP.

** Key words:**Lichen planus, biological markers, cytokines, enzyme-linked immunosorbent assay, immunosuppressive agents.

## Introduction

Lichen planus (LP) is a chronic inflammatory disease that affects skin and mucosa (1). Oral forms of lichen planus (OLP) are more common, chronic and recalcitrant than cutaneous type causing pain and discomfort leading to significant impairment of quality of life (2).

Because LP is a result of T cell mediated immune response, T cells locally present in the involved epithelial and subepithelial tissues (3) release proinflammatory cytokines including interleukin-6 (IL-6) and interleukin-8 (IL-8) (4,5). Levels of interleukins in the serum and saliva in OLP are considered a reliable indicator of therapeutic response on molecular basis (6-8). Also IL-6 has growth factor properties, suggesting its role in the development and progression of cancer (8).

Keratinocytes in OLP present major histocompatibility complex (MHC) class II antigens to CD4+ T cells causing secretion of Th1 cytokines interleukin-2 and interferon-γ. These cytokines in turn activate the CD8+ T cells causing keratinocyte apoptosis via tumour necrosis factor- α (TNF- α). Also TNF-α enhances the activation of nuclear factor-κB in the subepithelial T cells and as a consequence leads to the increased expression of other proinflammatory cytokines including IL-6 (4,5). IL-6 has been associated with multidrug resistance protein (MRP) expression by the keratinocytes (9) and has been demonstrated by some to play a role in the oral cancer pathogenesis (7,10).

This disease probably represents a cell mediated immunologic response to an induced antigenic change in the mucosa of predisposed individuals (1). Thus the use of immunomodulators or immunosuppressants to control the disease would appear reasonable. Although, Corticosteroids have been the mainstay of treatment for OLP since long, but there is no evidence of long term remission being established. Further corticosteroids are also associated with their own side effects (11).

Calcineurin inhibitors have been tried with good results in cases which are recalcitrant to corticosteroids and they have lesser side effects (12-14). Pimecrolimus 1% cream has been approved by the FDA for Atopic dermatitis (15). It has been suggested to be effective in the management of symptoms and erosions of OLP (12,16,17). Pimecrolimus has a similar mode of action to that of Tacrolimus but is more selective with no effect on dendritic cells (15) and has been associated with better efficacy and side effect profile than Tacrolimus (12-14). Mycophenolate mofetil (MMF) has been tried with high success rates in recalcitrant cases of erosive OLP (EOLP) and long term remission with safer side effect profile compared to other immunosuppesive drugs like Cyclosporine, Azathioprine and Corticosteroids has been reported (18). Methotrexate (MTX) as a supplement with topical steroids also has shown success in resolving erosive OLP cases recalcitrant to other common treatment modalities (19,10). To date there are few published studies regarding the efficacy of Pimecrolimus (17,20,21) and its comparison with other therapies in OLP (14) and fewer are available for MTX (20,21) and MMF in OLP (18,22-24) and have a limited sample size. We have conducted a large scale study with 42 EOLP patients.

As IL-6 has been associated with MRP expression by the keratinocytes with up regulation of MRP expression seen in the lichen planus lesions (9); hence we intended to evaluate the effects of various drugs on serum IL-6 and correlation of these effects with the nature of clinical response, alongwith the resistance pattern seen in OLP lesions with various therapeutic modalities. Thus we evaluated the role of serum IL-6 in deciding therapy for multidrug resistant OLP. As of now, most of the studies with these drugs in OLP have focused on the clinical aspects of the disease only. But broad based interventions are required at molecular level to assess the pathways involved in resistance to drugs and the therapeutic response.

## Material and Methods

The proposed study was approved by the institutional ethical committee of Maulana Azad Institute of Dental Sciences (MAIDS); and was conducted in the department of Oral Medicine and Radiology, MAIDS, New Delhi, in association with department of Biochemistry, Maulana Azad Medical College. This study was a prospective, double blind, randomised clinical trial. Patients were selected from the outpatient department of MAIDS; and those with histologically proven erosive oral lichen planus (EOLP) were included in the study. Patients selected were between the 19 and 72 years old, since OLP is an unusual disease in childhood. Pa-tients were excluded from the study in cases of Lichenoid contact reaction due to any medication, mouthrinse, toothpaste or any other agent; any therapy for Lichen Planus or drugs associated with Lichenoid reaction within past 8 weeks; any malignant or viral involvement in mouth; pregnant or nursing women also excluded, patients not willing to participate in the study, patients whose complete follow up was lacking. 

Patient selection was done based on the characteristic clinical features of OLP (Bilateral reticular striae with atrophic erosive areas). Informed consent regarding the procedures being performed was taken from the patients selected. Pretreatment Biopsy from the representative area was done, for the confirmation of diagnosis with the characteristic histopathological findings of subepithelial dense lymphocytic infiltrate, basilar vacuolization with apoptosis leading to homogeneous eosinophilic civette bodies formation. Serum samples of the confirmed cases were collected for Enzyme linked immunosorbent assay (ELISA) to determine baseline serum levels of IL-6 (pg/ml).

In this study 42 EOLP patients were enrolled. Mean age at presentation of EOLP was 46.95±11.96 (19-72) years. Mean duration since when the lesions have been present was 21.293±20.77 (1-246) months. Male to female ratio was 8:7. Along with negative control group of 10 normal mucosa, of patients undergoing minor surgery as well as healthy volunteers; and positive control group of 10 histopathologically proven Oral squamous cell carcinoma (OSCC) cases was selected for comparison of serum IL-6 levels, which were age and sex matched with the OLP group.

All the OLP cases were randomised into 3 treatment groups, each with 14 subjects. These groups were subjected to 3 different types of treatments as 1) Pimecrolimus, 2) Mycophenlate Mofetil (MMF) with Pimecrolimus and 3) Methotrexate (MTX) with Pimecrolimus. All the patients were advised to stop any other drug treatment if taking for at least 8 weeks. Pimecrolimus was prescribed as 1% (w/w) topical aqueous gel form, four times daily, for 9 months, after meals and patient was advised not to drink and eat for at least ½ hour after topical application. MMF was prescribed as oral 1 gram daily dosase in 2 equally divided doses for 3 to 4 months depending upon the clinical response and 0.5 gram as once daily dosage for next 3 months; and patient was advised to take the drug empty stomach (1 hour before food or 2 hours after meals). MTX was prescribed as 7.5 mg weekly dosa-ge for 7-9 months depending upon the response. Patient was advised to take the drug empty stomach and to take with food if gastrointestinal disturbances occurred and was advised to avoid taking milk rich products alongwith drug. Laboratory tests were performed for estimation of liver function, kidney function, haemoglobin (Hb) level, platelet count at beginning of treatment in all the groups; and at a regular interval of 15 days, in both the groups subjected to systemic treatment with MMF and MTX. Platelet count below 1 lakh and Hb levels below 10 mg/dl was considered an indication to taper the drugs. Post- treatment serum samples were collected for serum IL-6 estimation at the end of six months of treatment, in each case.

-Collection of samples: Approximately 2 mL of blood was collected by venipuncture and placed into a serum separator tube. The blood sample was allowed to clot for 30 minutes before processing the serum. After centrifugation for 10 minutes at approximately 3000g, the serum was decanted into a new 5-mL vial and stored at -70ºC until analyzed.

-Estimation of serum IL-6 levels (ELISA): IL-6 concentration in the collected serum samples was determined by ELISA using commercially obtained ELISA kits (KRISHGHEN BioSystems). Briefly the procedure was as follows: (1) IL-6 working standard and dilution series prepared. (2) 100 μL of assay diluents added to each well (3) 100 mL of standard or sample added and incubated for 2 hours; (4) 200 mL of IL-6 conjugate added and incubated for 2 hours; (5) 200 mL of substrate solution added and incubated for 20 minutes; (6) 50 mL of stop solution added. Data were read at 450/550 nm (EL 808; Biotek Instrument, Winooski, VT) and results were expressed as pg/mL. Photon output was considered proportional to IL-6 concentration and IL-6 concentration was being evaluated from standard curve.

Comparison of serum IL 6 levels pre and post treatment along with comparison among different groups (EOLP Vs. Negative control, EOLP Vs. Positive control) and subgroups (Treatment groups- pre Vs. Post-treatment and among various treatment groups) has been done.

-Statistical analysis plan: Because some variables in the study groups were not normally distributed, nonparametric statistics were applied. Only two-sided probability values less than .05 were considered as significant. The data were entered in software (SPSS 13 for windows, SPSS Inc, Chicago, III). Continuous data were reported as mean ± SD. A Kruskal-Wallis test was carried out for multiple comparisons, while Mann-Whitney tests were performed to detect a difference between each pair of groups in rank sign. Intra group comparison of various parameters at different points of time (Pre and post treatment difference of various markers) was done by using Wilcoxon signed rank test.

## Results

Interleukin 6 levels were found to be raised above 3pg/ml (upper limit of normal range detected in this study) in only 26.19% of the EOLP cases with mean of 3.72±8.14. IL6 levels of EOLP were found to be significantly different from negative control (*P*<0.05- Mann-Whitney U test). Pre and post-treatment levels were significantly different in MMF group only (*P*<0.05- Mann-Whitney U test). Serum IL6 levels in OSCC were not found to be elevated above 3pg/ml ( Table 1).

**Table 1:**
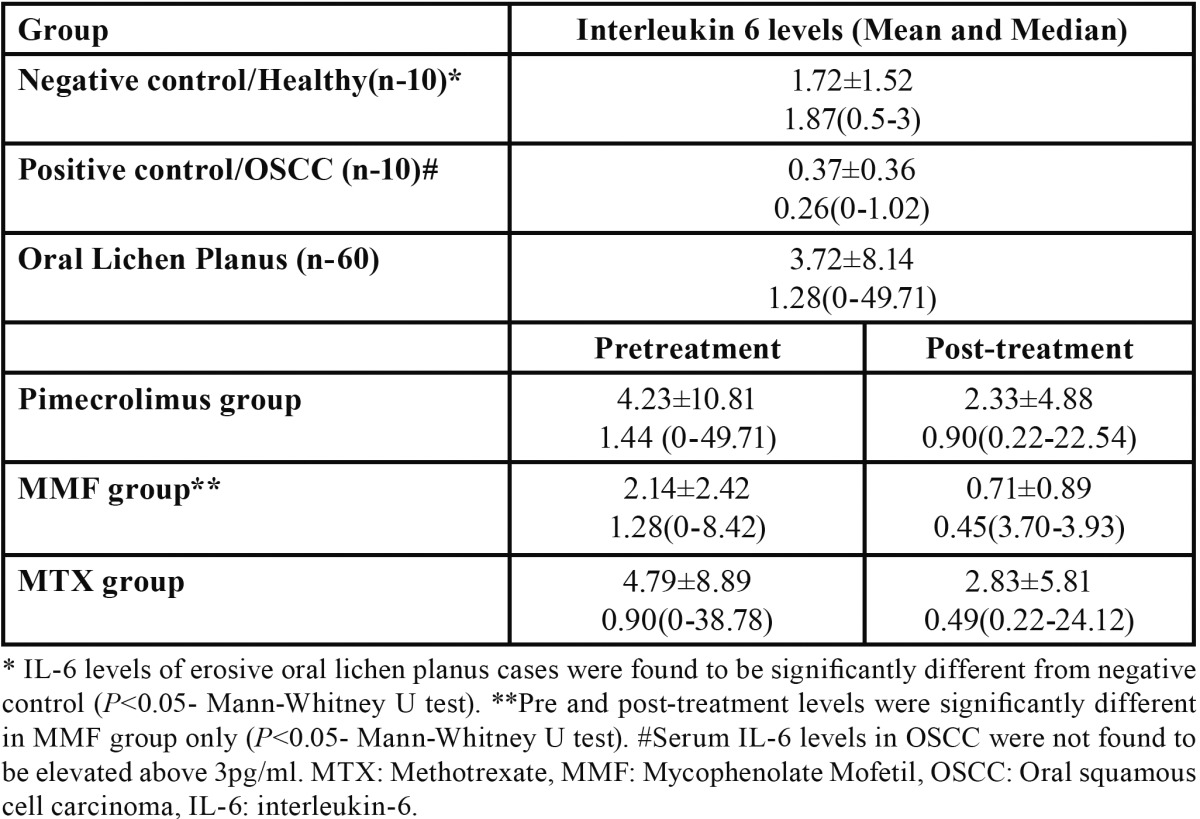
Serum IL-6 levels (pg/ml).

By Wilcoxon signed rank test a significant decrease was noted in serum IL6 levels after treatment in MMF group; while no significant decrease in serum IL6 levels was noted in Pimecrolimus and Methotrexate groups ( Table 2). In this study 5% of the EOLP cases with IL-6 levels above 5pg/ml were resistant to treatment with MTX supplemented with Pimecrolimus. In a case with IL-6 levels of 6.45 pg/ml, eruption of skin lesions as well as aggravation of oral lesions was noted while on MTX therapy which did not resolve even after continued treatment with MTX for 45 days.

**Table 2:**
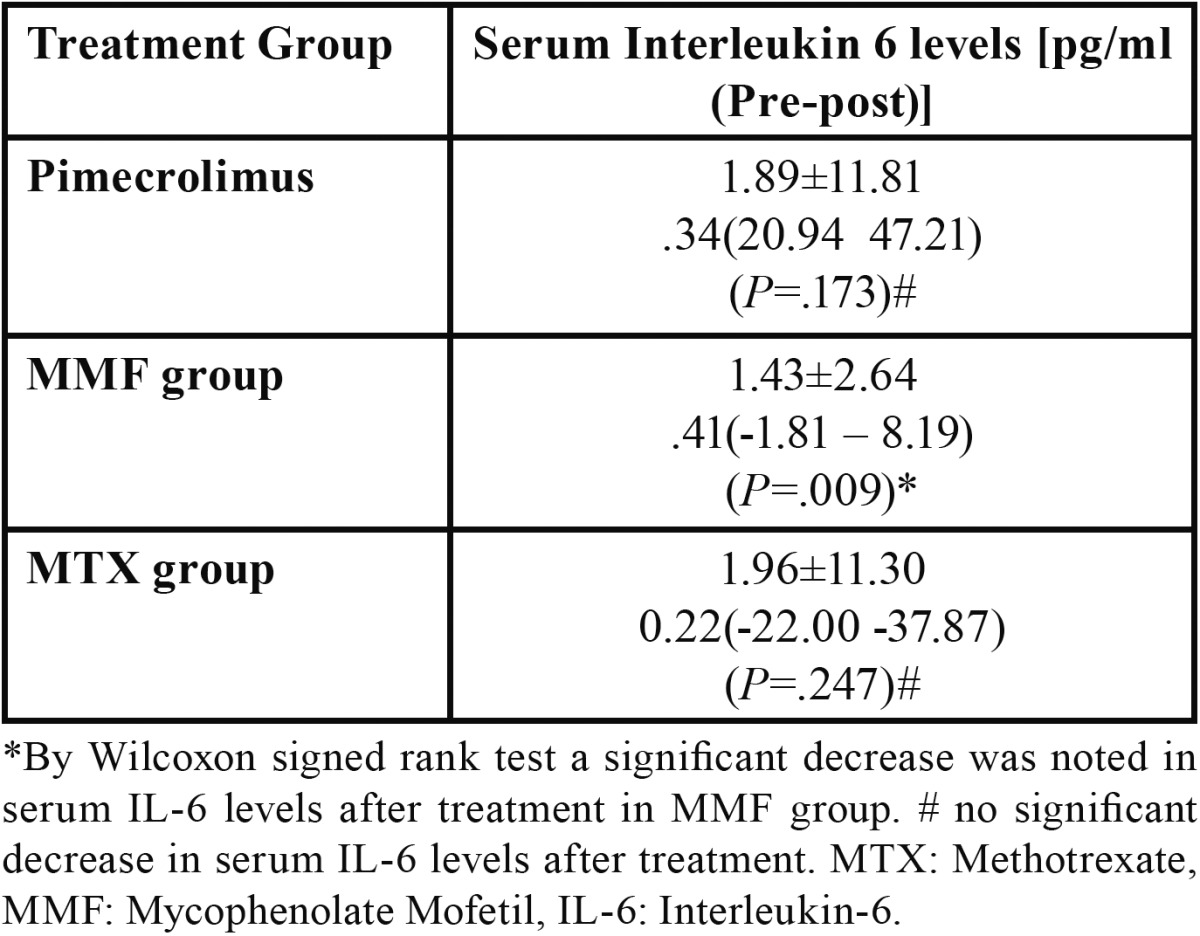
Difference pre to post-treatment IL6.

-Side effect profile 

Regarding safety profile a significant decrease in platelet count was seen in some patients in MTX group at 3-4th months period requiring tapering of the drug for a period of about 15 days-1 month based upon restoration of count although there was no significant change in liver and kidney function profile and there was no gastrointestinal upset during use. MMF was well tolerated in all the cases and there was no complaint of nausea, vomiting and diarrhoea and kidney and liver function profile was stable. Although transient burning sensation was noted with Pimecrolimus during application in initial 15-20 days of treatment but it was not a hindrance to treatment and patient compliance.

## Discussion

IL-6 levels were found to be raised in only 26.19% of the EOLP cases (mean: 3.72±8.14 pg/ml) compared to normal mucosa (mean: 1.72±1.52) in our study; which was statistically significant (*P*<0.05). This finding is supported by previous studies, where significantly increased serum IL-6 levels were noted in OLP (*P*<0.05) (6,24); where raised serum IL-6 levels were reported by Sun *et al.* (6) in only 15% of the EOLP cases (mean 3.4±3.2 pg/ml) and by Yamamoto *et al.* (24) in 50% of EOLP cases with IL-6 levels above 40 pg/ml in some cases. This finding reflects the chronic inflammatory nature of the disease (25). Although raised serum IL-6 levels in OLP have also been suggested to reflect its malignant potential (7).

Lichen planus is assumed to represent a delayed hypersensitivity reaction, in the course of which cytokines, including IL-6, control the proliferation and differentiation of cytotoxic T lymphocytes which attack the epidermis and cause apoptosis of the undifferentiated keratinocytes (26).

IL-6, a pleiotropic cytokine with varied systemic functions, plays a major role in inflammatory processes as a host immune defense mechanism (27). However, increased or deregulated expression of IL-6 significantly contributes to the pathogenesis of various human diseases including OLP. Numerous preclinical and clinical studies have revealed the pathological roles of the IL-6 pathway in inflammation, autoimmunity, and cancer. Based on the rich body of studies on biological activities of IL-6 and its pathological roles, therapeutic strategies targeting the IL-6 pathway are in development for cancers, inflammatory and autoimmune diseases (28).

In our study a significant decrease was noted in serum IL-6 levels after treatment in MMF group (*P*=0.009) ( Table 2). This may suggest the involvement of IL-6 in EOLP as shown by the previous studies (4,9,26) and targeting of IL-6 pathway by MMF with a possible role of IL-6 in assessing the therapeutic response. Nagy *et al.* (29) reported a significant decrease in production of IL-6 after MMF intervention, which accords with our findings. Similar decrease in IL6 with other treatments was noted in other studies (6,7).

Decrease in IL-6 levels may have a significant role to play in clinical resolution of the sign and symptoms associated with OLP as it has been found that stimulation of the normal human epidermal keratinocytes and primary human dermal fibroblasts with IL-6 results in an upregulation of MRP expression and activity (9). Correspondingly, upregulation of MRP expression was found in the lesional skin taken from the patients with lichen planus (9).

In this study 5% of the OLP cases have shown serum IL-6 levels above 5pg/ml and were resistant to MTX therapy; finding sup-ported a previous study, where resistance to MTX was reported (20). These findings may necessitate the finding of promising alternative treatments for patients with multidrug resistance predicted with measurement of serum IL-6 levels having a possible role in guiding the therapy.

Serum IL-6 levels in OSCC were not found to be elevated ( Table 1), finding supported by a previous study (24); although serum IL-6 has been found to be raised in other body cancers where anti IL-6 monoclonal antibody therapy has been useful in the treatment of cancer (27). Contrary to our finding some clinical studies have reported increase in serum IL-6 levels in OSCC (7). Thus further studies are required to establish the role of IL-6 in OSCC and so in OLP to evaluate its malignant potential in this context.

So, comparison of various therapeutic modalities for OLP regarding their effects on serum IL-6 levels reveals that there is a marked possibility for these markers to help decide the best possible therapy for a case, which after this study is proposed to be MTX supplemented with Pimecrolimus and a possible role of MMF supplemented with topical therapy in cases recalcitrant to MTX.

## Conclusions

Significantly raised serum IL-6 levels in OLP reflect the chronic inflammatory nature of the disease and that serum IL-6 can be a possible biomarker in deciding the best possible therapy for treatment resistant EOLP and in therapeutic monitoring. However further studies are required to establish the role of IL-6 in OSCC and so in OLP to evaluate its malignant potential in this context.
